# Case report: A case of acute exacerbation of interstitial pneumonia associated with TAFRO syndrome

**DOI:** 10.3389/fmed.2023.1137899

**Published:** 2023-09-07

**Authors:** Yoshitaka Shimada, Yasushi Nagaba, Mako Fujino, Hiroyuki Okawa, Kaori Ehara, Eri Shishido, Shinya Okada, Hiroaki Yokomori

**Affiliations:** ^1^Department of Nephrology, Kitasato University Medical Center, Kitamoto, Japan; ^2^Department of Rheumatology, Kitasato University Medical Center, Kitamoto, Japan; ^3^Department of Pathology, Kitasato University Medical Center, Kitamoto, Japan; ^4^Department of Internal Medicine, Kitasato University Medical Center, Kitamoto, Japan

**Keywords:** TAFRO syndrome, interstitial pneumonia, acute exacerbation, diffuse alveolar damage, interleukin-6, cytokine storm

## Abstract

Cytokine storm caused by the overproduction of inflammatory interleukin (IL)-6 plays a central role in the development of acute inflammation. The extremely rare disease, TAFRO syndrome, progresses quickly. Renal dysfunction, fever, reticulin fibrosis, anasarca, thrombocytopenia, and organomegaly with pathological findings such as idiopathic multicentric Castleman disease are all characteristics of TAFRO syndrome. Interstitial pneumonia (IP), which is not characteristic of this disease, is probably a complication of the inflammatory process. An 88-year-old man presented with a 3-day history of fever, dry cough, and progressive dyspnea. After he was first treated with antibiotics, he was transferred to our hospital because he showed no improvement. Data showed hemoglobin Hb 90.00 (SI) (9.0 g/dL); leukocyte count WBC 23 × 10^9^/L (SI) [23,000/μL (neutrophils 87.5%, lymphocytes 2.5%, blast cells 0%)]; hemoglobin 90 g/L (9.0 g/dL); platelet count 101.00 × 10^9^/L (10 100/μL); lactate dehydrogenase 4.78 μkat/L (286 U/L); serum albumin 25.00 g/L (2.5 g/dL); blood urea nitrogen 18.17 μmol/L (50.9 mg/dL); creatinine 285.53 μmol/L (3.23 mg/dL); C-reactive protein 161.50 mg/L (16.15 mg/dL); IL-61830 pg/mL; and surfactant protein D level 26.6 ng/mL. Findings from computed tomography indicated increased ground-glass opacities without traction bronchiectasis consistent with acute IP. The diagnosis was leukocytosis and progressive kidney injury. After bone marrow aspiration caused by persistent pancytopenia, mild reticulin fibrosis was identified. Because of the high IL-6 concentration, which revealed small atrophic follicles with regressed germinal centers surrounded by several lymphocytes, right inguinal lymph node biopsy was performed. Two minor and three major criteria led to diagnosis of TAFRO syndrome. Administrations of antibiotic therapy and methylprednisolone pulse therapy were ineffective. After rapid progress of respiratory failure, the patient died on day 30 of hospitalization. Autopsy of lung tissues showed diffuse alveolar damage with hyaline membranes. Based on these findings, we diagnosed acute exacerbation of IP associated with TAFRO syndrome due to IL-6 overproduction-associated cytokine storm.

## 1. Introduction

Cytokine storm is an umbrella term encompassing several disorders with immune dysregulation. It is characterized by the presence of constitutional symptoms, systemic inflammation, and multiorgan dysfunction that can engender multiorgan failure if treated inadequately. Prominent elevation of serum inflammatory cytokine concentrations, such as those of interferon-γ (or CXCL9 [chemokine {C-X-C motif} ligand], CXCL10, or chemokines induced by interferon-γ), interleukin (IL)-10, IL-6, and soluble IL-2 receptor alpha is typical. The latter is a marker of T-cell activation (1).

Takai et al. (2) were the first to report concurrent thrombocytopenia, anasarca, fever, reticulin fibrosis, and organomegaly as TAFRO syndrome in 2010. Extreme generalized edema, designated as anasarca, is a medical condition including effusion of fluid into the extracellular space, causing swelling of the skin (3). The sometimes fatal systemic inflammatory TAFRO syndrome has acute to subacute onset. It is regarded as a unique clinicopathological variant of multicentric (M) Castleman disease (CD), a rare lymphoproliferative disorder. Histopathologically the disease has been subclassified into four types by Castleman Disease Collaborative Network classification: plasmablastic, plasma-cell, hyaline-vascular, and mixed. Moreover, it has been subclassified clinically into MCD and unicentric CD and (4). The MCD pathogenesis involves dysregulated cytokine activity, which causes lymphadenopathy and systemic inflammatory symptoms (3, 4). TAFRO syndrome patients have higher concentrations of vascular endothelial growth factor and IL-6 in sera and effusion, but the disease pathogenesis remains unclear (3). Typically, these patients have severe forms of cytokine storm (1, 5). However, most researchers have recently thought that TAFRO syndrome differs from typical iMCD (6). Moreover, some patients with TAFRO syndrome do not demonstrate lymphadenopathy. Therefore, some aspects of the clinical and histological manifestations of iMCD and TAFRO syndrome may overlap, but TAFRO is not included in iMCD. iMCD-TAFRO might be a part of iMCD (6).

Diffuse parenchymal lung disorders with highly variable clinical courses and poor outcomes are considered collectively as interstitial lung diseases, despite their heterogeneity (7). Particularly interstitial pneumonia (IP) is a heterogeneous group of non-neoplastic diffuse parenchymal lung diseases that result from damage to the lungs by varying degrees of inflammation and fibrosis (8). With the variable clinical courses of these diseases, acute exacerbation (AE) represents a life-threatening condition with remarkable morbidity and high mortality (8–11).

Consistent findings on computed tomography (CT) of patients with TAFRO syndrome include ascites, mesenteric edema, retroperitoneal edema, and pleural effusion. Moreover, high rates of gall bladder wall edema, periportal collar, pericardial effusion, and subcutaneous edema are found among patients (12). To date, no TAFRO patients have shown evidence of honeycombing, traction bronchiectasis, reticular opacities, architectural distortion, and volume loss, with or without ground-glass opacities (2, 3). This report describes a rare case of AE of IP with features of TAFRO syndrome with hyper-IL-6 syndrome.

## 2. Case description

**Figure 1:**
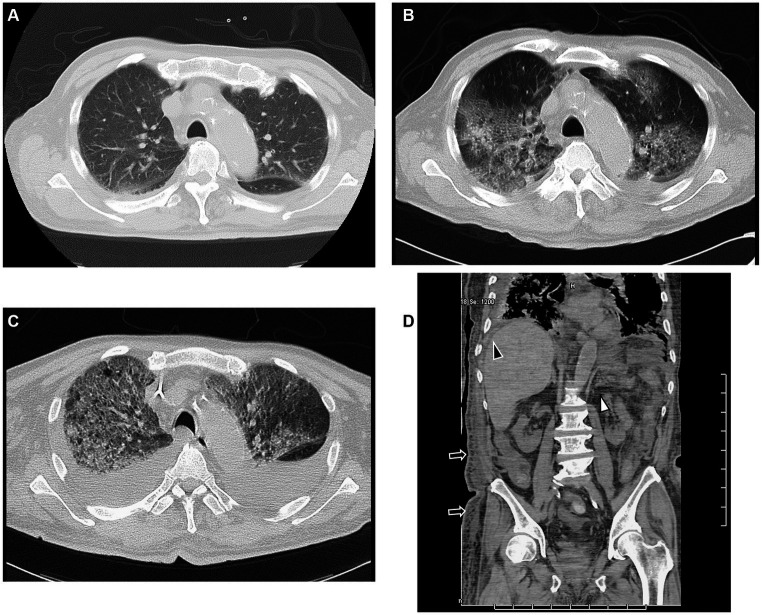
CT findings. **(A)** CT scan shows slightly infiltrated consolidation in the lower lung lobes on admission. **(B)** CT scan shows acute interstitial pneumonitis showing diffuse ground-glass opacity in both lungs and posterior consolidation in the right lung evidenced by air bronchograms with ascites on the fourth day of hospitalization. **(C)** CT scan shows rapid progression of consolidation and ground-glass opacity with ascites on the 12th day of hospitalization. **(D)** Sagittal CT scan shows ascites (black arrowhead), mesenteric edema (white arrowhead), and subcutaneous edema (black arrow), but no lymphadenopathy on the fourth day of hospitalization. CT, computed tomography.

A previously healthy 89-year-old man was brought to the emergency department of the outside hospital (OSH) after falling on the floor, without loss of consciousness. He had a 3-day history of sore throat and lethargy associated with mild cough. He denied the presence of fever, chills, or shortness of breath. Upon admission, he was afebrile, without tachypnea or oxygen desaturation on room air. Pathological workup included negative results of respiratory cultures for viral and atypical pneumonia pathogens, blood cultures, and urine *Streptococcus* and *Legionella*. He received penicillin, cephalosporins, and azithromycin at the OSH. After 3 days, he was admitted to our hospital in an emergency as he was gradually developing renal dysfunction. On admission, his vital signs were the following: temperature 39°C, pulse rate 110 beats/min, respiratory rate 18 breaths/min that increased to 28 breaths/min when the patient’s general condition worsened, blood pressure 131/75 mmHg, and oxygen saturation 86% on room air. Chest auscultation revealed increased breath sounds with crackles in the bilateral lower lobes. The remainder of the physical examination was unremarkable. Chest CT showed bilateral nonspecific cellular IP, which tends to be a dominant feature: it can be distributed symmetrically or diffusely in a basal predominance (Figure 1A).

**Figure 2:**
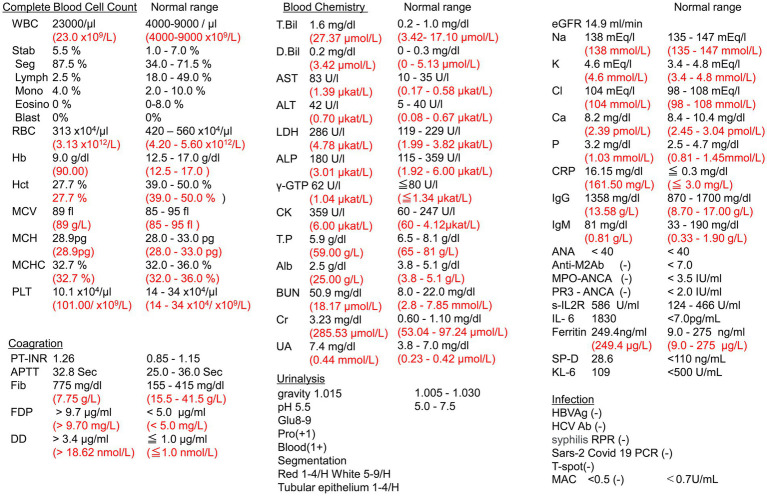
Laboratory data. WBC, white blood cell; RBC, red blood cell; PLT, platelet; PT-INR, prothrombin time/international normalized ratio; APTT, partial thromboplastin time; Fib, fibrin; FDP, fibrin/fibrinogen degradation products; DD, Ddimer; T-Bil, total bilirubin; D-Bil, direct bilirubin; AST, aspartate aminotransferase; ALT, alanine aminotransferase; γ-GTP, glutamyl transpeptidase; ALP, alkaline phosphatase; LDH, lactate dehydrogenase; CK, creatine kinase; T.P, total protein; Alb, albumin; BUN, blood urea nitrogen; Cr, creatinine; UA, urine acid; eGFR, estimated glomerular filtration rate; Na, sodium; K, potassium; Cl, chloride; Ca, calcium; NH3; ammonia; CRP, C-reactive protein; IgG, immunoglobulin G; IgM, immunoglobulin M; ANA, anti-nuclear antibody; Anti-AMA M2Ab, anti-mitochondrial M2 antibody; MPO-ANCA, myeloperoxidase perinuclear antineutrophil antibody; PR3-ANCA, proteinase 3 cytoplasmic antineutrophil antibody; Soluble IL2R, soluble interleukin-2 receptor; IL-6, interleukin-6; HBV Ag, hepatitis B antigen; HCV Ab; hepatitis C Ab; T-spot, T-spot TB; MAC, *Mycobacterium avium*-*intracellulare* complex; RPR, syphilis rapid plasma regain; SP-D, surfactant protein D; KL-6, Krebs von den Lungen 6. SI unit is shown in red.

Results of laboratory investigations were the following: hemoglobin 90.00 (SI) (9.0 g/dL); MCV 89 fl; leukocyte count 23.0 × 10^9^/L [23,000/μL (neutrophils 87.5%, lymphocytes 2.5%, blast cells 0%)]; platelet count 101.00 × 10^9^/L (10 100/μL); total protein 59.00 g/L (5.9 g/dL); serum albumin 25.00 g/L (2.5 g/dL); lactate dehydrogenase 4.78 μkat/L (286 U/L); creatinine 285.53 μmol/L (3.23 mg/dL); blood urea nitrogen 18.17 μmol/L (50.9 mg/dL); s-IL2R 586 U/mL, IL-61830 pg/mL; C-reactive protein (161.50 mg/L (16.15 mg/dL); and surfactant protein D level 26.6 ng/mL); fibrin/fibrinogen degeneration products 9.70 mg/L (9.7 μg/mL); Ddimer 18.62 nmol/L (3.4 μg/mL); negative COVID-19 RT PCR; and negative T-SPOT TB test. The autoimmune antibody profile and tumor biomarkers were within normal limits (Figure 2).

During his clinical course, the patient was febrile with tachypnea and showed increased leukocyte count and decreased platelet count despite antibiotic therapy (tazobactam/piperacillin), which was switched to meropenem. Urinary tests showed a Urea-protein (1+) concentration of 0.58 g/g•Cr, which was considered low, and a serum creatinine concentration of 3.23 mg/dL. Urine output decreased to 500–600 mL/day, leading to the diagnosis of oliguria. Because vital signs indicated a pre-shock state and his respiratory condition was unstable, the patient was diagnosed with sepsis when he was admitted to our hospital, and continuous renal replacement therapy (CRRT) was started for sepsis. Next, the urine output was maintained, serum creatinine concentration tended to improve, and CRRT was discontinued. Thereafter, CRRT was restarted, but it was difficult to manage body fluids even when using diuretics as urine output decreased again. Nevertheless, because sepsis was suspected again, CRRT was restarted.

On hospital day 4, CT showed increased ground-glass opacities and bilateral parenchymal consolidation predominantly at the lung bases with pleural effusion (Figure 1B). We then reassessed the clinical symptoms and pathological data. He had thrombocytopenia, anasarca, fever, renal dysfunction, and a serum IL-6 level of 1830 pg/mL Moreover, the patient’s platelet count decreased, and his leukocyte count increased (platelets 3,400/μL and leukocytes 59,000/μL) on the eighth day of admission. We then conducted a bone marrow biopsy.

**Figure 3:**
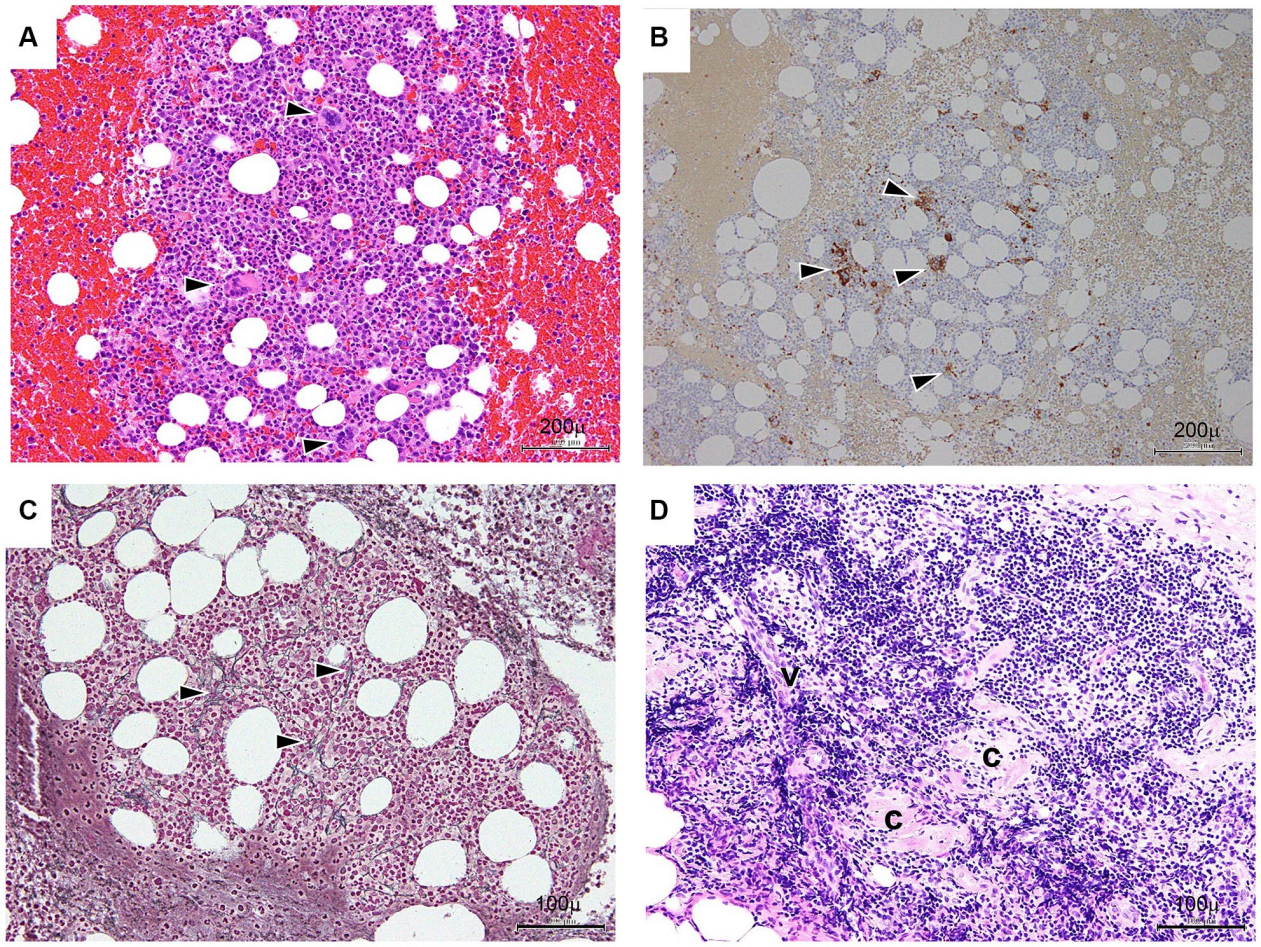
Bone marrow **(A–C)** and lymph node **(D)** findings. **(A)** Bone marrow trephine (hematoxylin and eosin staining) – normocellular with increased megakaryocytes. Arrowheads indicate a megakaryocyte. Bar: 200 μm. **(B)** Immunohistochemical CD41b on the bone marrow trephine biopsy shows increased megakaryocytes. Arrowheads indicate reaction products of megakaryocytes. Bar: 200 μm. **(C)** Bone marrow trephine (reticulin) – consistent with grade 1 fibrosis (black arrows). Bar: 100 μm. **(D)** Lymph nodes in the inguinal fossa show small atrophic follicles (C) with regressed germinal centers surrounded by several lymphocytes. Occasional vessels (V) penetrating the germinal centers are seen. Bar: 100 μm.

Bone marrow (BM) aspiration was performed to exclude the presence of malignant tumors. The results showed normocellular BM with increased megakaryocytes (Figures 3A,B) and fibrosis with a positive reticulin stain (MF-1) (Figure 3C) (13). In addition, the biopsy of the lymph nodes in the right inguinal fossa showed small atrophic follicles with a regressed germinal center surrounded by several lymphocytes. Occasional vessels penetrating the germinal centers were also noted (Figure 3D). Based on these patterns of histological expression, a diagnosis of CD, hyaline-vascular type, was made (3).

On hospital day 8, the major findings were anasarca, thrombocytopenia, and high CRP level, while the minor findings were reticulin myelofibrosis and renal insufficiency, thereby leading to the diagnosis of TAFRO syndrome (1).

**Figure 4:**
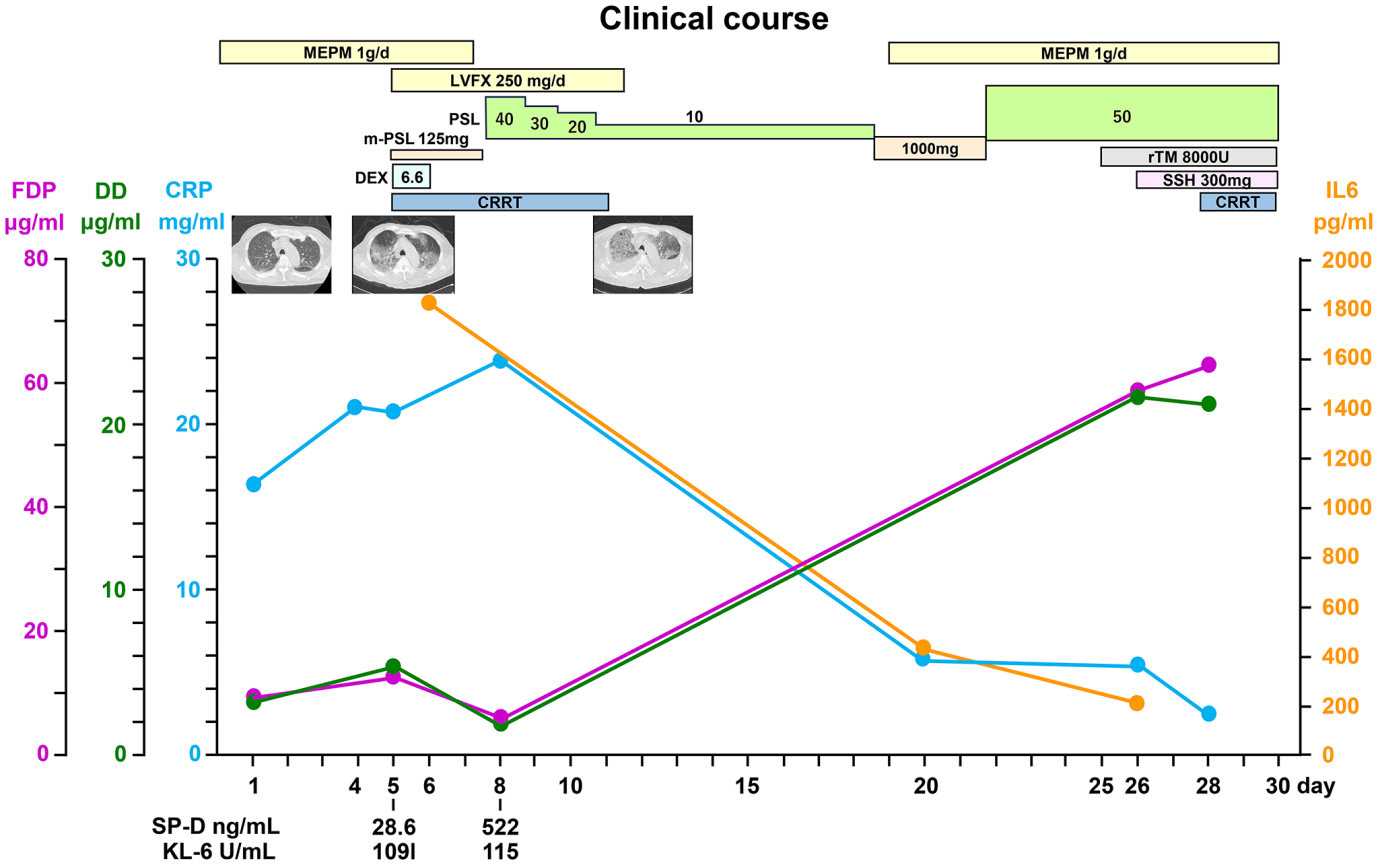
Clinical course. LVFX, levofloxacin; IL-6, interleukin-6; SP-D, surfactant protein D; KL-6, Krebs von den Lungen 6; FDP, fibrin/fibrinogen degradation products; DD, Ddimer; CRP, C-reactive protein; PSL, prednisolone; CRRT, continuous renal replacement therapy; rTM, thrombomodulin alfa; SSH, sivelestat sodium hydrate.

The patient underwent methylprednisolone pulse therapy and antibiotic therapy. These interventions were effective to improve CRP and IL-6 levels (Figure 4), but respiratory failure progressed rapidly (Figures 1C, 4). The decreased platelet count and hemoglobin level and increased Ddimer level indicated a diagnosis of disseminated intravascular coagulation. Ultimately, the patient expired on hospital day 26 due to severe hypoxemia with progressive lung involvement (Figure 4).

**Figure 5:**
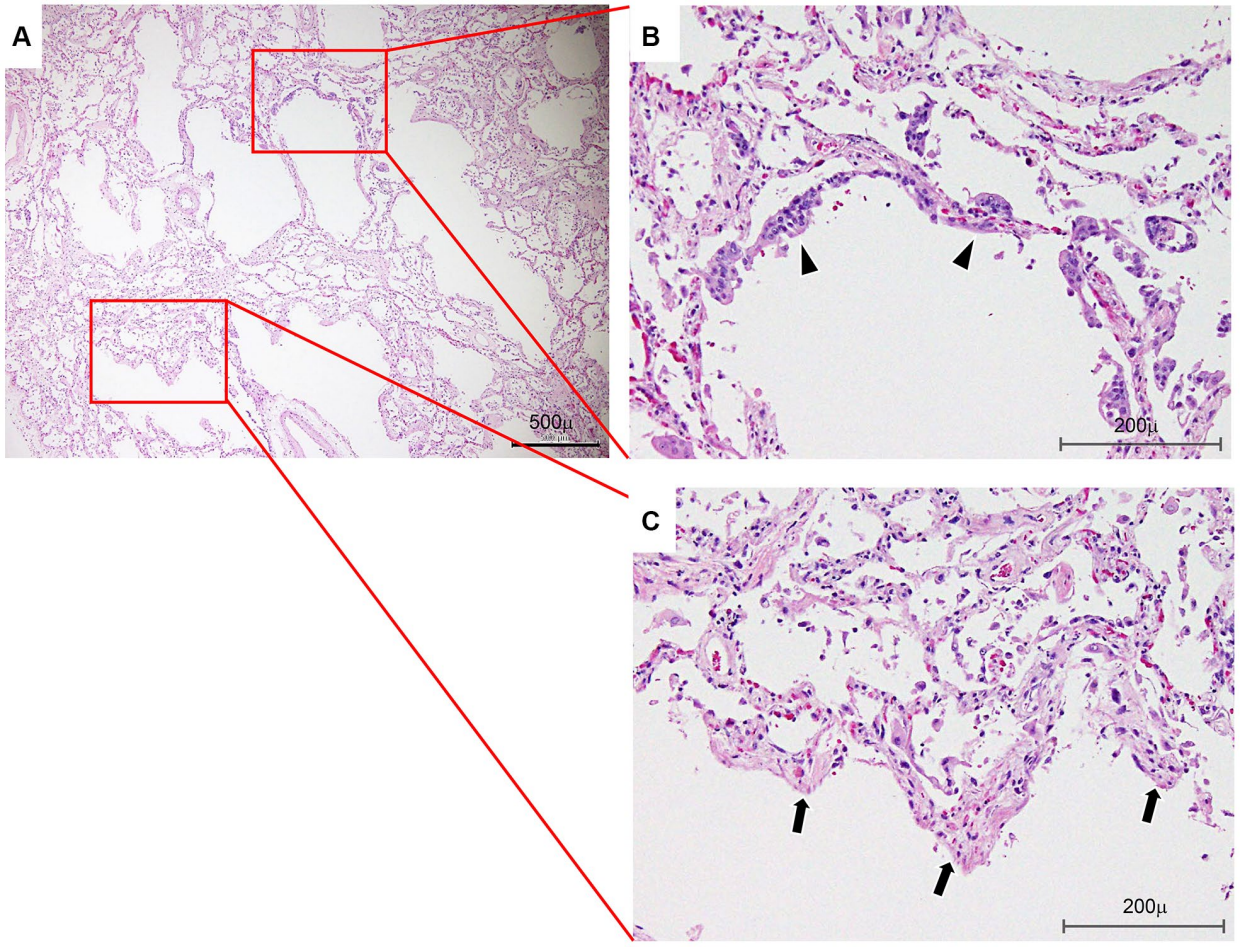
Lung histology. **(A)** Photomicrograph showing an area of associated diffuse alveolar damage at a lower magnification. Bar: 500 μm. **(B)** Photograph showing squamous metaplasia of bronchiolar epithelium (arrowheads). Bar: 200 μm. **(C)** Note hyaline membranes lining the alveolar ducts (arrows) and partially collapsed adjacent alveoli (hematoxylin and eosin staining) (arrows). Bar: 200 μm.

We obtained informed consent for the publication of this case report from the family of the patient. Histological findings at autopsy revealed DAD, with hyaline membranes and squamous metaplasia of bronchiolar epithelium (Figures 5A–C) superimposed on UIP. Renal pathology at autopsy identified nephrosclerosis as the underlying cause. However, glomerular lesions were scarce, and there were no findings strongly suggestive of acute interstitial nephritis. This suggested renal injury due to renal artery thrombosis. Regarding liver pathology at autopsy, microscopic observation of the liver showed necrosis of hepatocytes from Zone 3 to Zone 2 of the hepatic lobule as well as cholestasis. Based on these findings, hepatic ischemia was confirmed despite normal histology of the liver.

## 3. Diagnostic assessment

The major findings were anasarca, thrombocytopenia, and high CRP level, while the minor findings were reticulin myelofibrosis and renal insufficiency, leading to the diagnosis of TAFRO syndrome (3). Moreover, AE of IP is regarded as an indistinguishable histopathological pattern of DAD from the histologic pattern (7–9, 12–14). We diagnosed IP associated with TAFRO syndrome due to IL-6 overproduction-associated cytokine storm (1).

## 4. Discussion

This case of acute IP with TAFRO syndrome is the first ever reported. The onset of symptoms in most patients with acute IP is usually gradual, with progressive shortness of breath, dry cough, and dyspnea being the most common presentation (6, 7). However, lung involvement in idiopathic MCD (iMCD) commonly engenders poorly defined centrilobular nodules, thin-walled cysts, bronchovascular bundle thickening, and interlobular septal thickening. These findings have been categorized as a lymphocytic IP pattern in iMCD (4). According to clinical differences between iMCD and TAFRO syndrome, TAFRO syndrome patients were found to have significantly higher rates of mortality, renal failure, anasarca, and fever with and without iMCD than in patients with iMCD. Findings also indicate that TAFRO syndrome patients with and without iMCD had significantly higher concentrations of Ddimer, creatinine, glutamyl transpeptidase, alkaline phosphatase, blood urea nitrogen, CRP, and fibrin/fibrinogen degradation product. However, TAFRO syndrome patients with and without iMCD were found to have significantly lower total protein and albumin concentrations and platelet counts than in iMCD patients (3).

We believe that the development of DAD with usual interstitial pneumonia (UIP), called interstitial pulmonary fibrosis (IPF)-AE, corresponds to acute respiratory distress syndrome of any etiology in IPF and should be managed accordingly (12–14).

IL-6 trans-signaling components indirectly cause the release of transforming growth factor beta (TGF-β), which is a pro-fibrotic growth factor that can influence pathway activation and disease progression. This suggests the importance of IL-6 in the pathogenesis of IPF (1, 11). Cytokine storm and hyper-IL-6 syndrome occur in this condition.

AE is a rapidly progressive form of IP that develops in an otherwise healthy individual over a few days to weeks. It is characterized by the histologic finding of DAD. In the case presented in this paper, CT showed the presence of ground-glass opacity without consolidation, which results from DAD in the acute phase (12–14).

Previously studied plasma biomarkers in acute lung injury include Krebs von den Lungen-6 (KL-6) and surfactant protein D (SP-D), which is a marker of type II cell injury and/or proliferation (13). Cases of IPF-AE have been reported with normal KL-6 level at the time of AE diagnosis (14).

In the current case, despite the presence of diffuse ground-glass opacity on CT and DAD on histopathology, the KL-6 level was normal, while the SP-A level increased. In patients with IPF-AE, organizing DAD that is superimposed on UIP indicates a poor prognosis despite a normal KL-6 level at the time of AE diagnosis (15).

The TAFRO syndrome onset is acute in most cases. It progresses rapidly. It is fatal without urgent treatment. For disease severity assessment, points are assigned for several items: (1) thrombocytopenia, (2) renal insufficiency, (3) fever/inflammation, and (4) anasarca (including pleural effusion and ascites) (2, 3, 5, 16). This report describes a rare case of acute interstitial pneumonia (AIP) with the features of TAFRO syndrome (17).

In the case described herein, no superficial lymphadenopathy was evident on CT (Figure 1D) because of the obscurity of the target superficial lymph nodes. We investigated the inguinal lymph nodes via biopsy. The TAFRO syndrome diagnostic criteria which are used in Japan have included patients without histologically confirmed of lymph node involvement (2, 16). In TAFRO syndrome patients, lymph node involvement is often slight or nonexistent. Conducting biopsies is difficult because of severe anasarca and bleeding tendencies attributable to thrombocytopenia (16). However, we performed lymph node biopsy using an inguinal lymph node specimen. After examining lymph nodes in the inguinal fossa region, we found small atrophic follicles with regressed germinal centers surrounded by several lymphocytes. Occasional vessels penetrating the germinal centers were also seen.

Patients with MCD, TAFRO syndrome (1, 3, 5, 14), and IPF-AE (8–10) have all been found to have hyper-IL-6 syndrome. Elevated IL-6 concentrations might be found in patients who have other diseases, which renders elevated IL-6 unsuitable for differential diagnosis. To elucidate these disorders, identification must be made of novel TAFRO syndrome disease-specific biomarkers.

## Data availability statement

The original contributions presented in the study are included in the article/supplementary material, further inquiries can be directed to the corresponding author.

## Ethics statement

The studies involving humans were approved by the Ethics Committee of Kitasato University Medical Center. The studies were conducted in accordance with the local legislation and institutional requirements. The participants provided their written informed consent to participate in this study. Written informed consent was obtained from the participant/patient(s) for the publication of this case report.

## Author contributions

YS, YN, HO, KE, MF, ES, and HY designed the case report and drafted the manuscript. YS and SO contributed to the review of this manuscript. All authors read and approved the final manuscript.

## Conflict of interest

The authors declare that the research was conducted in the absence of any commercial or financial relationships that could be construed as a potential conflict of interest.

## Publisher’s note

All claims expressed in this article are solely those of the authors and do not necessarily represent those of their affiliated organizations, or those of the publisher, the editors and the reviewers. Any product that may be evaluated in this article, or claim that may be made by its manufacturer, is not guaranteed or endorsed by the publisher.

## Abbreviations

TAFRO: Thrombocytopenia, Anasarca, Fever, Reticulin fibrosis, Organomegaly
iMCD: Idiopathic multicentric Castleman disease
TP: Total protein
UA: uric acid
FDP: Fibrin/fibrinogen degradation products
DD: Ddimer
SP-D: Surfactant protein D
KL-6: Krebs von den Lungen 6
IPF: idiopathic interstitial fibrosis
DAD: diffuse alveolar damage
AE: acute exacerbation
AIP: acute interstitial pneumonia
UIP: usual interstitial pneumonia
